# The Safety of Open Surgical Tracheotomy Performed by Otorhinolaryngology Residents

**DOI:** 10.12669/pjms.343.14907

**Published:** 2018

**Authors:** Burak Ulkumen, Gorkem Eskiizmir, Onur Celik

**Affiliations:** 1 Burak Ulkumen, MD. Assistant Professor, Manisa Celal Bayar University, Department of Otorhinolaryngology-Head Neck Surgery, Manisa, Turkey; 2 Gorkem Eskiizmir Associate Professor, Manisa Celal Bayar University, Department of Otorhinolaryngology-Head Neck Surgery, Manisa, Turkey; 3 Prof. Onur Celik, Manisa Celal Bayar University, Department of Otorhinolaryngology-Head Neck Surgery, Manisa, Turkey

**Keywords:** Complication, Open surgical tracheotomy, Otorhinolaryngology residency, resident

## Abstract

**Objective:**

We aimed to clarify the safety of open surgical tracheotomy performed by supervised residents, and the impact of “reason for hospitalization” on complication rates in open surgical tracheotomy technique.

**Methods:**

In this retrospective cohort study, the medical files and documents of 277 patients who underwent open surgical tracheotomy (OST) over a period of 12 years from October 2005 to July 2017 were analyzed. Forty four patients were excluded due to emergent tracheotomy and presence of malignancy. Remaining 223 cases were divided into two groups as “OSTs done by supervised residents” and “OSTs done by attending surgeons”. Age, gender, reason for hospitalization, observation time and complications were noted. The overall minor and major complication rates and each complication rate were compared with regard to the operating surgeons.

**Results:**

No statistically significant difference between two groups was demonstrated in terms of observation time (*p*=0.127). Minor complication rate for residents and attending surgeons was 14.7% and 17.5%, whereas major complication rate was 6.3% and 5.0%, respectively. No significant difference was found between two groups both in terms of minor (*p*=0.58) and major (*p*=0.43) complication rates. No risk of “reason for hospitalization” on minor and major complications was found (*p*=0.06, *p=*0.15).

**Conclusion:**

Open surgical tracheotomy performed by supervised residents is as safer as the ones performed by the attending surgeons. The study also showed that “reason for hospitalization” does not potentiate the occurrence of tracheotomy related complications.

## INTRODUCTION

Open Surgical Tracheotomy (OST) has long been used mainly for securing upper airway. Although, the term “tracheotomy” was first used in 1739 by Lorenz Heister, it dates back to 2000 BC.^1^ Currently, two main indications for tracheotomy are Upper Airway Compromise (UAC) and Prolonged Intubation (PI).^2^ In the first half of the 20th century, tracheotomies performed due to UAC were far more frequent with indications of infections such as diphtheria, acute supraglottitis and deep neck abscesses. However, PI recently gets ahead because of novel preventive measures for infectious diseases and widespread use of mechanical ventilation.^3^

OST is known as a relatively safe procedure and it is one of the initial interventions learned in otorhinolaryngology residency program. Never the less, it may lead to some complications.^2^,^4^ Minor complications have been reported as; hemorrhage without any surgical intervention, subcutaneous emphysema, keloid, decannulation, wound infection while major complications as; hemorrhage that required surgical intervention, decannulation with a high risk of airway failure, pneumothorax requiring chest tube insertion, esophagotracheal fistula, tracheomalacia, tracheoinnominate artery fistula and death due to tracheotomy.^4^,^5^ Thus, performing OST by residents, early in the training program, may cause concerns from the point of possible complications.

There are lots of studies comparing the complication rates of attending surgeons and residents^6^-^9^ concerning different residency programs other than Otorhinolaryngology. In light of these studies, there is still no consensus on whether the interventions done by residents have higher complication rates or not. There is only one study evaluating the safety of OST performed by residents. In this study Fiorini et al. Compare overall complication rates without classifying them into minor and major and they found no higher overall complication rate in OSTs performed by residents.^10^ We aimed to find out if there is any difference between OSTs performed by supervised residents and attending surgeons in terms of minor and major complication rates, which is not studied before. We also evaluated the effect of Reason for Hospitalization (RfH) on complication rates.

## METHODS

In this retrospective cohort study, the medical files and documents of 277 patients who underwent OST over a period of 12 years from October 2005 to July 2017 were analyzed. Cases that underwent OST were determined from our institutional archiving software. Next, detailed data were obtained from hard copy files of these cases. The exclusion criteria were as follows: (i) presence of malignant neoplasm of aero-digestive tract and (ii) emergency tracheotomy. Thirty-two patients with a previous malignant neoplasm of larynx, hypopharynx or oral cavity and 22 patients who underwent emergency tracheotomy were excluded. Thereby, 223 eligible cases were enrolled into the study. Then, the remaining cases were divided into two groups as Group I (OSTs done by supervised residents) and Group II (OSTs done by attending surgeons).

This research received approval from the institutional review board. A sample size of 184 cases was determined based on a power of 95% with an effect size of *α*_2_ = 0.05.

### Influential factors and complications

Age, gender, RfH, observation time, minor and major complications were noted. Minor complications were defined as: (i) hemorrhage without any surgical intervention, (ii) subcutaneous emphysema, (iii) keloid, (iv) decannulation, (v) wound infection while major complications as: (i) hemorrhage that required surgical intervention, (ii) decannulation with a high risk of airway failure, (iii) pneumothorax that required chest tube insertion, (iv) esophagotracheal fistula, (v) tracheomalacia, (vi) tracheoinnominate artery fistula and (vii) death due to tracheotomy.

### Surgical procedure

**Fig.1:**
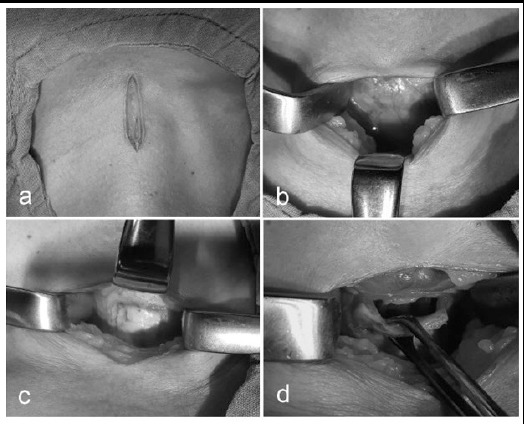
Surgical steps of open surgical tracheotomy. (a) Vertical skin incision (b) After dissection of strap muscles, thyroid isthmus is seen (c) Tracheal flap incision by unipolar cautery (d) Elevation of inferiorly based tracheal flap.

All OSTs were performed in the operating room. A vertical incision was used in all cases (Fig.1a). Following blunt dissection of the strap muscles (Fig.1b) and retraction of thyroid isthmus, an inferiorly based tracheal flap was created at the level between the 2^nd^ and 3^rd^ tracheal rings (Fig.1c). This flap was preferably sutured to the skin for ease urgent re-cannulation in case of unintentional decannulation (Fig.1d). Cannulas with high volume low pressure cuffs were used for cannulation.

### Statistical analysis

The overall minor and major complication rates and each complication rate were compared as regard the operating surgeons. The data were presented as mean ± SD and Shapiro-Wilk test was used for the assessment of distribution. The comparisons of quantitative data between groups were made using independent samples t-test or Mann Whitney U test according to the results of normality test. The impact of RfH on minor and major complication rates was evaluated by cox multivariate analyses. The results in confidence interval of 95% and *p*<0.05 were considered statistically significant (IBM SPSS Statistics for Windows, Version 21.0.; Armonk, NY, IBM Corp).

## RESULTS

### Descriptive statistics

A total of 223 cases were enrolled into the study. Hundred and forty-three patients [(72 females, 71 males); mean age ± Standard Deviation (SD) 52.07±16.68] underwent OST by residents whereas the remaining 80 cases [(45 females, 35 males); mean age ± Standard Deviation (SD) 57.14±22.50] underwent OST by attending surgeon. No statistically significant difference between two groups was demonstrated in terms of age (*p*=0.364) and sex (*p*=0.398).

Two main indications for tracheotomy were prolonged intubation (PI) (unable to wean from mechanic ventilation) (196 patients, 91.6%) and Upper Airway Compromise (UAC) (27 patients, 12.1%). The clinical data about the distribution of tracheotomy indications and the reasons for hospitalization, according to the operating surgeons is presented in Table-I. There were no statistically significant differences in the distribution of indications except “cerebrovascular disorder” (Table-I).

**Table-I:** The distribution of tracheotomy indications and the reasons for hospitalization according to the operating surgeons

Reason for hospitalisation	(Resident) n (%)	(Surgeon) n (%)	(Total) n (%)	*P* value
***Prolonged intubation***				
Cerebrovascular Disorder	57 (25.6)	68 (30.5)	125 (56.1)	0.01
Cardiovascular disease	3 (1.3)	16 (7.2)	19 (8.5)	0.06
Pneumonia/exacerbation of COPD	10 (4.5)	21 (9.4)	31 (13.9)	0.65
Other neurological diseases	4 (1.8)	12 (5.4)	16 (7.2)	0.35
Trauma and/or intoxication	1 (0.5)	2 (0.9)	3 (1.3)	0.93
Major surgery	--------	2 (0.9)	2 (0.9)	0.29
**Total**	75 (26.7)	121 (64.9)	196 (91.6)	0.58
**Upper airway compromise**				
Maxillofacial trauma	3 (1.3)	13 (0.6)	16 (7.2)	0.14
Laryngotracheal trauma	1 (0.5)	2 (0.9)	3 (1.3)	0.09
Bilateral choanal atresia	1 (0.5)	2 (0.9)	3 (1.3)	0.93
Deep neck infection	--------	3 (1.3)	3 (1.3)	0.92
Congenital laryngeal anomaly	--------	1 (0.5)	1 (0.5)	0.19
Angioedema	--------	1 (0.5)	1 (0.5)	0.45
**Total**	5 (2.2)	22 (9.9)	27 (12.1)	0.43
**Overall**	80 (35.9)	143 (64.1)	223(100)	

*Percentages are given according to overall population of 223 patients.

Observation time for Group-I and Group-II were; 335.14±195.20 (range 67-850 days) and 294.33±188.63 (range 22-848 days), respectively. No statistically significant difference between two groups was demonstrated in terms of observation time (*p*=0.127).

### Complications

Minor complication rates for residents and attending surgeons were 14.7% and 17.5%, whereas major complication rates were 6.3% and 5.0%, respectively. No significant difference was found between two groups both in terms of minor and major complication rates. Moreover, no statistically significant differences were found between two groups for each single complication when analyzed individually. The most common minor complication for both groups was “*hemorrhage*” while there was no explicit preponderance of any major complication (Table-II). Risk analyses revealed no effect of RfH on both minor and major complications (Table III and IV).

**Table-II:** The comparison of complication rates according to the operating surgeons

Type of Complication	Resident n (%)	Surgeon n (%)	p value
**Minor**			
Hemorrhage	7 (4.9)	6 (7.5)	0.43
Subcutaneous emphysema	4 (2.8)	2 (2.5)	0.90
Keloid	1 (0.7)	---	0.45
Decannulation	4 (2.8)	3 (3.8)	0.70
Wound infection	5 (3.5)	3 (3.8)	0.92
**Total**	21 (14.7)	14 (17.5)	0.58
**Major**			
Major hemorrhage with surgical intervention	2 (1.4)	---	0.29
Decannulation with a risk of airway failure	1 (0.7)	2 (2.5)	0.26
Pneumothorax with chest tube insertion	2 (1.4)	---	0.29
Esophagotracheal fistula	3 (3.8)	---	0.19
Tracheomalacia	---	1 (1.3)	0.18
Other life-threatening events	1 (0.7)	---	0.46
Death due to tracheotomy	2 (2.5)	1(0.7)	0.93
**Total**	9(6.3)	4 (5.0)	0.42

*Percentages are given in subgroup basis (Resident and Surgeon).

**Table-III:** Impact of reasons for hospitalization on minor complication rates.

RfH	HR	p value	95% CI
**Prolonged intubation**			
Cerebrovascular Disorder	1.04	0.99	1.40-3.34
Cardiovascular disease	0.05	0.96	0.90-2.56
Pneumonia/exacerbation of COPD	0.05	0.95	0.50-2.40
Other neurological diseases	0.53	0.93	0.67-2.45
Trauma and/or intoxication	1.26	0.88	0.20-4.05
Major surgery	1.21	0.75	1.20-2.89
**Total**	0.42	0.02	0.10-1.43
**Upper airway compromise**			
Maxillofacial trauma	0.00	0.95	1.30-4.40
Laryngotracheal trauma	1.77	0.87	0.80-2.55
Bilateral choanal atresia	0.65	0.65	1.25-3.45
Deep neck infection	0.45	0.90	0.19-1.80
Congenital laryngeal anomaly	1.12	0.99	0.20-3.78
Angioedema	0.18	0.80	0.46-1.25
**Total**	2.36	0.06	1.05-5.31

HR: Hazard ratio, CI: Confidence interval.

**Table-IV:** Impact of reasons for hospitalization on major complication rates

RfH	HR	p value	95% CI
**Prolonged intubation**			
Cerebrovascular Disorder	0.08	0.97	0.30-2.34
Cardiovascular disease	0.85	0.99	0.57-1.76
Pneumonia/exacerbation of COPD	0.03	0.96	0.20-3.40
Other neurological diseases	0.05	0.96	0.01-1.35
Trauma and/or intoxication	0.96	0.50	1.23-7.65
Major surgery	0.95	0.34	0.98-1.55
**Total**	0.39	0.15	0.10-1.43
**Upper airway compromise**			
Maxillofacial trauma	0.01	0.08	1.30-4.40
Laryngotracheal trauma	0.63	0.45	0.80-2.55
Bilateral choanal atresia	0.00	0.99	1.25-3.45
Deep neck infection	0.99	0.95	0.19-1.80
Congenital laryngeal anomaly	0.56	0.88	0.20-3.78
Angioedema	0.78	0.95	0.46-1.25
**Total**	2.59	0.15	0.60-9.63

HR: Hazard ratio, CI: Confidence interval.

## DISCUSSION

The majority of our study population consists of patients who underwent tracheotomy because of PI (91.8%). RfH in patients with PI were predominantly cerebrovascular disorders and cardiovascular diseases in both groups. This is in agreement with similar studies.^11^,^12^ We also evaluated the difference between Group I and Group II in terms of distribution of RfH and found significant difference only in cardiovascular disease (Table-I).

Evaluation of safety for a particular surgical intervention can be done by determining the incidence of complications and risk factors. In this retrospective cohort study we determined the incidence of minor and major complications and compared them from the point of performer (resident, attending surgeon). There have been plenty of studies evaluating the safety of particular surgeries done by residents as part of different residency programs. However there is still no consensus on whether the operations are safe at least as the ones done by attending surgeons.^6^-^9^ There is one study evaluating “percutaneous dilatational tracheotomy” as part of the otorhinolaryngology residency program but they reported a relatively high mortality rate (3 of 21 cases).^13^ In the current study mortality rate was 1.35%. When it comes to OST, which is among the initial surgical procedures trained in otolaryngology residency, there is only one study evaluating the safety in terms of performer.^10^ In that study, Fiorini et al. Compare the overall complication rates between supervised residents and surgeons. But the study groups were not homogenous, namely they did not exclude emergency cases and cases with malignancy of upper aero-digestive tract which might led bias. Besides they did not report if there is any statistically important difference in the distribution of these aforementioned cases on groups’ basis. Unlike, we exclude emergent OSTs and OSTs with upper aero-digestive tract malignancies to preclude bias. Another problem with that study was that the mechanically ventilated group of patients was unequally distributed between resident and surgeon groups which also make the results contradictory. In the current study all indications were equally distributed on group’s basis except “cerebrovascular disorder” (Table-I). Even though according to risk analyses, impact of cardiovascular disorder was found neither on minor nor on major complication rates (Table III and IV). Thus heterogeneity of cardiovascular disorders between groups can be ignored.

In some studies complications were categorized according to time of procedure as perioperative and postoperative (short term, long term). For example, Glysen et al. found higher short term complication rates but lower long term complication rates for percutaneous dilatational tracheotomy.^11^ Fiorini et al. also categorise them as perioperative, early and late.^10^ In the current study, complications were not categorized according to time of procedure. Because, to make an inference about safety, one should consider eventual complication rates rather than relying on timeline.

In this study, alongside overall complication rates, all complications were analyzed individually. And, statistically significant difference was not found in any single minor or major complication rate. Besides, none of the cases had a tracheoinnominate artery fistula which is a rare and lethal complication.^14^ The main reported risk factors for this complication are low surgical level, placement of cannulas with high pressure cuff and direct trauma by the cannula due to extreme head tilt.^15^ The absence of this complication may be related to cannulas with high volume low pressure cuffs which have been used in our institution.

The incidence of minor and / or major complications has not been analyzed individually according to the operating surgeons in any previous study. Our results demonstrated that minor complication rate was slightly high while major complication rate was slightly low in Group II, but these differences were statistically insignificant. In brief, “OST performed by supervised residents” is as much safer as the “OST performed by the attending surgeon”. We also showed that RfH does not potentiate the occurrence of tracheotomy related complications.

## CONCLUSIONS

It is safe and appropriate for supervised residents to perform OST at the initial period of otorhinolaryngology residency program. RfH does not have any effect on both minor and major complication rates of OST.

### Author’s Contribution

**BU, GE:** Conceived, designed and did statistical analysis & editing of manuscript.

**BU, GE & OC:** Did data collection and manuscript writing.

**GE & OC:** Did review and final approval of manuscript.
